# A Novel β-adaptin/c-Myc Complex Formation Modulated by Oxidative Stress in the Control of the Cell Cycle in Macrophages and its Implication in Atherogenesis

**DOI:** 10.1038/s41598-017-13880-5

**Published:** 2017-10-18

**Authors:** Victor García-González, Jaime Mas-Oliva

**Affiliations:** 1 Instituto de Fisiología Celular, Universidad Nacional Autónoma de México, Ciudad de México, 04510 Mexico, Mexico; 2 Departamento de Bioquímica, Facultad de Medicina Mexicali, Universidad Autónoma de Baja California, Mexicali, Baja California 21000 Mexico

## Abstract

Our study tested the proposal that c-Myc activation in macrophages is differentially carried out dependent on the intracellular oxidative state of cells and potentially associated to the process of atherogenesis. Under our experimental conditions, the generation of reactive oxygen species carried out by the presence of oxidized low density lipoproteins (oxLDL) or Gram negative bacterial lipopolysaccharides (LPS) modifies the expression of cellular adhesion molecules such as c-Abl, calcium transport proteins such as the plasma membrane Ca^2+^-ATPase (PMCA), CD47, procaspase-7, CASP7, CHOP, transcriptional activators such as c-Jun and c-Myc and molecules that participate in the process of endocytosis like α- and β-adaptin. We present the first evidence showing that a state of oxidative stress alters c-Myc-dependent activity pathways in macrophages through binding to molecules such as β-adaptin promoting the reversible formation of a complex that presents the ability to regulate the development of the cell cycle. We propose that the subtle regulation carried out through the formation of this c-Myc/β-adaptin complex when cells change from a normal physiological condition to a state of oxidative stress, represents a defense mechanism against the deleterious effects caused by the loss of cell homeostasis.

## Introduction

Vascular endothelial damage considered a critical event during atherogenesis, usually progresses with the continuous accumulation of chemically modified lipoproteins in the sub-endothelium of blood vessels, the subsequent formation of chemically modified lipoproteins, the transformation of macrophage foam cells and the installment of tissue inflammation^1^. A critical event proposed during the early stages of the disease corresponds to the extravasation of monocytes into the sub-endothelium eventually differentiating into macrophages followed by a process of internalization of chemically modified low density lipoproteins such as oxidized (oxLDL), acetoacetylated (acLDL), carbamylated (cbLDL) or glycosylated (glLDL) lipoproteins. In consequence, the synthesis of pro-inflammatory molecules and the activation of cell death signaling pathways carried out in this cell type, contribute to the final establishment of atherosclerosis^2,3^.

Considering the role of endocytosis in down regulation of membrane receptors and cytoskeleton proteins, sophisticated mechanisms have been proposed where endocytosis through co-regulation of dual-function proteins might directly affect nucleus signaling pathways^4^. For instance, in addition to its role in endocytosis and signaling network modulated by phenomena such as cell growth and proliferation^5^, the nuclear involvement of adapter proteins such as eps15 and Clathrin Assembly Lymphoid Myeloid Leukemia Protein (CALM), have been reported as a positive modulator of transcription^6,7^. Likewise, several endocytic proteins have been directly described to regulate the transcriptional activity of p53, in turn modulating changes in its stability^8^.

Mainly based on experiments where inhibition of endocytosis does not modify the nuclear translocation of endocytic proteins and the blockage of nuclear protein export does not change the initial rate of endocytosis^6^, this process has been directly related to cell membrane phenomena, well known to be independent in relationship with processes associated to the nucleus-cytoplasmic shuttle. However, following the establishment of a state of cellular oxidative stress, this situation might be altered. Although it has been proposed that under specific physiological conditions several cellular antioxidant systems might be able to reduce oxidative stress^9^, reactive oxygen species (ROS) generated by enzymatic and non-enzymatic systems modifying lipids and sterols, produce their oxidized forms; if not controlled, triggering an inflammatory condition. In this sense, we have previously shown that a state of oxidative stress induced by treating cells with amyloid-like fibrils, triggers a series of perturbations in the expression of endocytic adaptor proteins^10–12^. We have also previously proposed that during oxLDL internalization, there is a critical role exerted by modified lipoproteins not only upon the expression of proteins involved in the process of endocytosis but also upon regulatory cell cycle proteins^13^. Therefore, direct changes in the capacity of cells to perform the process of endocytosis, might be also associated to mechanisms that modulate the cell cycle through proteins that present a dual function.

Therefore, the development of the present study has been focused on the description of a series of endocytic proteins showing this dual function, including the type of interactions that allow an effect upon the cell cycle. By exposing cells to oxLDL or bacterial LPS, we present a new role for β-adaptin showing the capability to interact and therefore to modulate the proto-oncogene c-Myc by forming a β-adaptin/c-Myc complex that controls cell cycle and viability in macrophages. Our results show that modulation of the endocytic process in this cell type is closely linked to an effect upon transport carried out between the nucleus and the cytoplasm. The detection of β-adaptin in an unexpected location such as the nucleus, together with the role it plays as part of the cytoskeleton, suggests that under specific conditions like a state of oxidative stress, β-adaptin could be considered a protein with dual properties.

## Results

**Figure 1:**
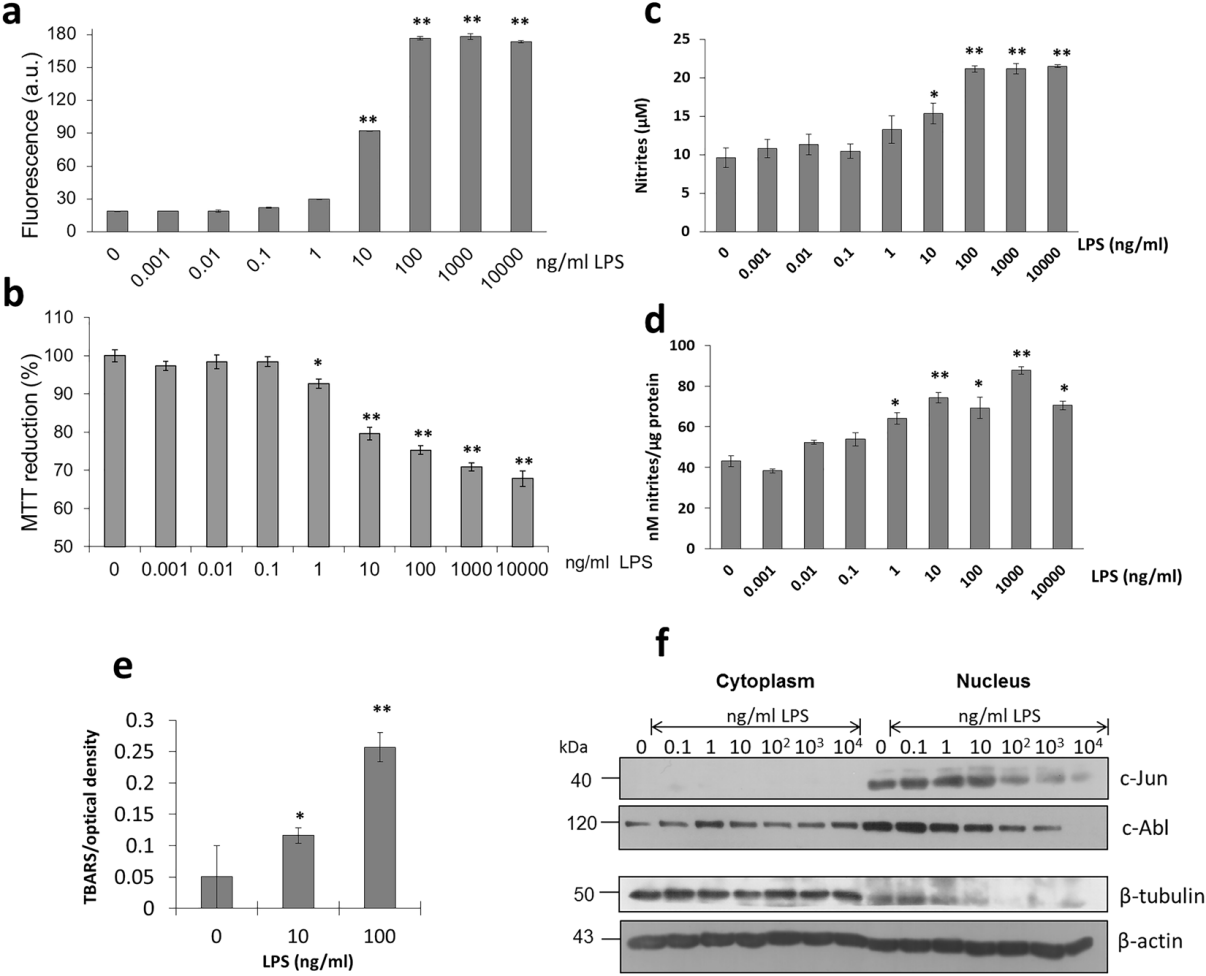
LPS promote transcriptional activity modifications in macrophages associated to a state of oxidative stress. (**a**) ROS detection by flow cytometry of macrophages treated with increasing amounts of LPS in the culture media for 16 h. (**b**) Cell viability assays by MTT. (**c**) Quantification of nitrites in the supernatant media of macrophages in culture after treatment with LPS for 12 h. (**d**) Determination of RNS in the cytoplasmic fraction of macrophages treated with increasing concentrations of LPS. (**e**) Determination of lipid peroxidation of macrophages treated with increasing LPS concentrations in the culture media using the TBARS assay. (**f**) Western blot detection of c-Jun and c-Abl in both the cytoplasmic and nuclear fractions of macrophages in response to the presence of increasing amounts of LPS in the culture media. β-tubulin was used as a control in a differentiated response between the nuclear and the cytoplasmic fractions while β-actin served as a general loading control. Mean values are presented (n = 5, X ± SD) *p < 0.05, **p < 0.01.

Macrophages stimulated with increasing concentrations of LPS (0–10^4^ ng/ml) generate an oxidative stress condition evaluated through the formation of ROS (Fig. 1a) and its impact upon cell viability (Fig. 1b).While LPS concentrations above 1 ng/ml seem to represent the threshold for ROS generation, at the same LPS concentration cell viability starts to be affected (Fig. 1b). LPS treatment (100 ng/ml) is comparable in magnitude with the amount of ROS generated when ter-butyl-hydroperoxide (400 µM) is employed (data not shown). Since reactive nitrogen species (RNS) produced by cells through the reaction of nitric oxide with superoxide to form peroxynitrite acts together with ROS to damage cells, we also evaluated cell nitrite production in response to LPS exposure. In the same fashion as found during ROS accumulation, at a threshold concentration of 1 ng/ml, LPS starts to significantly increase the amount of nitrites in both, cell culture media as well as in the cytoplasmic fraction of macrophages (Fig. 1c,d). Since ROS present very short half lives in comparison to RNS where nitrites can be readily measured, as a by-product of the activity promoted by ROS we evaluated lipid peroxidation by measuring thiobarbituric acid reactive substances (TBARS). Interestingly, we also found that LPS promote an important degree of cell lipid peroxidation with the presence of increasing amounts of LPS in the culture media (Fig. 1e).

Under the same experimental LPS concentrations, the macrophage nuclear and cytoplasmic fractions were separated and the analysis of transcription factor c-Jun and cell adhesion protein c-Abl quantitated together with β-tubulin (Fig. 1f). The nuclear expression of c-Jun decreases whenLPS treatment reaches 100 ng/ml, a phenomenon consistent with an increase in ROS and a decrease in cell viability (Fig. 1f). In parallel, c-Abl considered a negative regulator of cell migration through regulation of Crk-CAS complexes, was found also to decrease in the nuclear fraction with LPS concentrations above10 ng/ml, while in the cytoplasm c-Abl shows no changes in concentration (Fig. 1f). It has been proposed that a loss of control in this cascade may contribute to aberrant cell migration associated to inflammatory processes^13,14^. At low LPS concentrations (0.1, 1 and 10 ng/ml), an increased level of c-Jun is observed potentially associated to transcriptional activation of pro-inflammatory cytokines^15^, possibly in an initial attempt for cells to maintain homeostasis. Therefore, a decrease in the expression of these proteins associated to a diminution, for instance, in the nuclear concentration of β-tubulin in the nucleus (Fig. 1f), may be associated to one of the initial mechanisms of cell damage coupled to changes in the cytoskeleton and cell adhesion functions.

**Figure 2:**
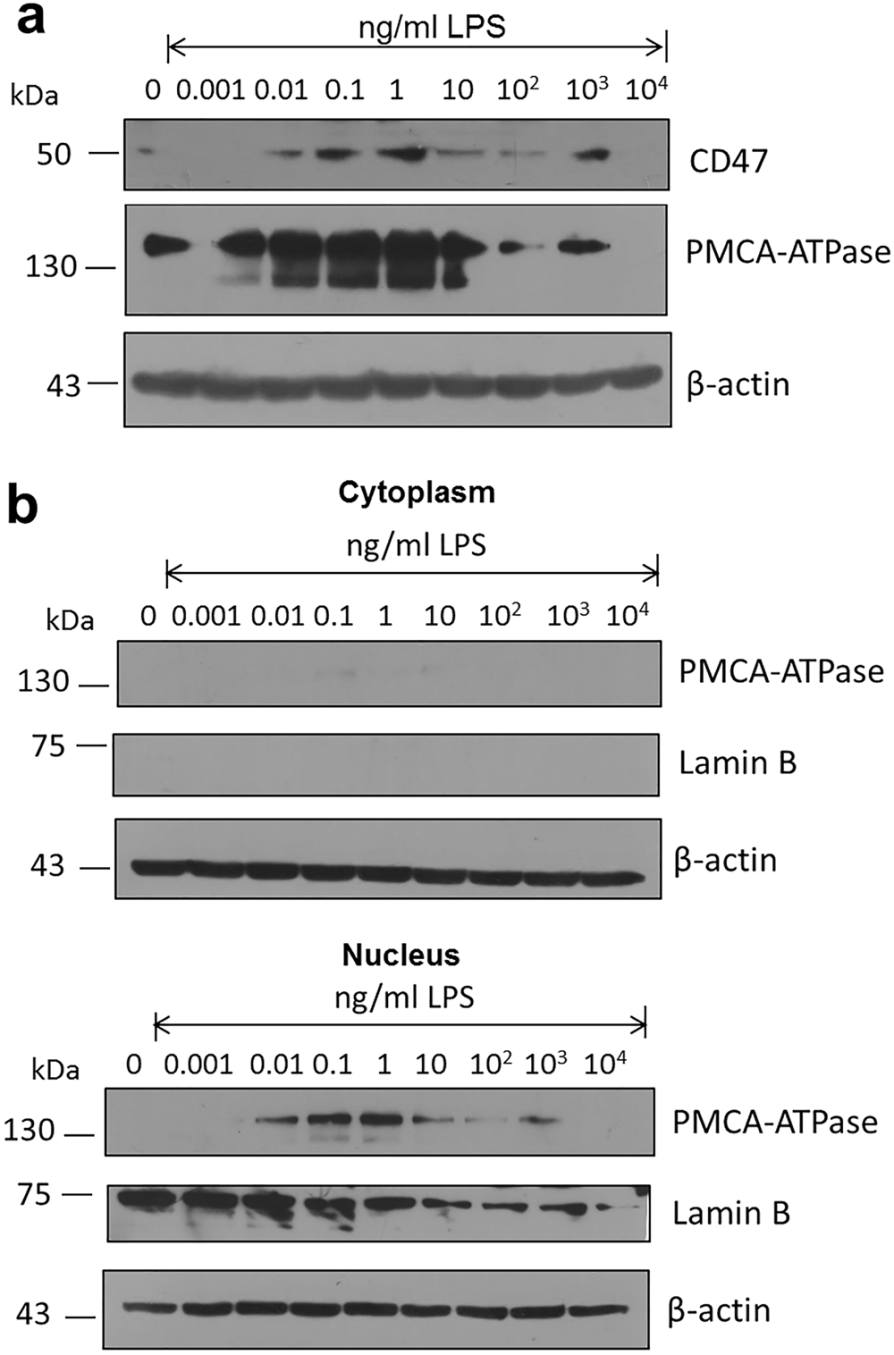
LPS treatment modifies the homeostasis of calcium in LPS treated macrophages by showing changes in the expression of both PMCA-ATPase and CD47 using western blot detection. (**a**) Expression levels of PMCA-ATPase and CD47 in total lysates of macrophages treated with increasing concentrations of LPS present in the culture media. (**b**) Expression levels of PMCA-ATPase and CD47 in the cytoplasmic and nuclear fractions obtained from macrophages treated with increasing concentrations of LPS present in the culture media. Lamin-B used as a control associated to the nuclear fraction while β-actin served as the general loading control.

Under these conditions, although the exact point of no return in terms of cell viability and proliferation is difficult to be determined, it has been recognized that the concentration of cytoplasmic calcium can be considered an important parameter in the initiation of irreversible cell changes when the concentration of this cation starts to increase; activating for instance, phospholipases, promoting the formation of eicosanoids and also triggering either a necrotic or an apoptotic phenomena. Interestingly, in our hands when the plasma membrane Ca^2+^-ATPase (PMCA), a transport protein with the function to remove calcium from the cell, is measured while macrophages are exposed to increasing amounts of LPS, the concentration of this protein in the total cellular fraction significantly increases when cells are exposed from 0.001 up to 1–10 ng/ml LPS, and drastically decreases with higher LPS concentrations (Fig. 2a). When separation of the cytoplasmic and nuclear fractions is carried out, PMCA is exclusively found in the nuclear fraction observing the same response to LPS (Fig. 2b). These series of experiments not only validate the effectiveness of our fractions separation, but define that macrophages under the adverse stimulus given by LPS respond by increasing the synthesis of this key protein in order to maintain at bay the cytoplasmic concentration of calcium up to a degree when viability importantly starts to decrease. The same effect is found when CD47, a high affinity receptor for thrombospondin-1 that selectively regulates integrin-dependent calcium fluxes is studied (Fig. 2a)^16–18^. As shown above for PMCA, the drastic decrease in concentration observed with CD47 corresponds to the same LPS concentrations where oxidative stress starts to develop and cell viability starts to be importantly affected (Fig. 1). Moreover, Lamin-B known to be involved in nuclear stability by being anchored to the inner surface of the nuclear membrane has been employed by us as a nuclear marker. Interestingly, Lamin-B also responds to high LPS concentrations by decreasing its presence exclusively in the nuclear fraction (Fig. 2b). Since one of the major targets for caspases is this family of proteins^19,20^, again the possibility for caspases to also participate in the origin of cell membrane perturbances and nuclear structure disarray, support the potential participation of an apoptotic phenomena.

**Figure 3:**
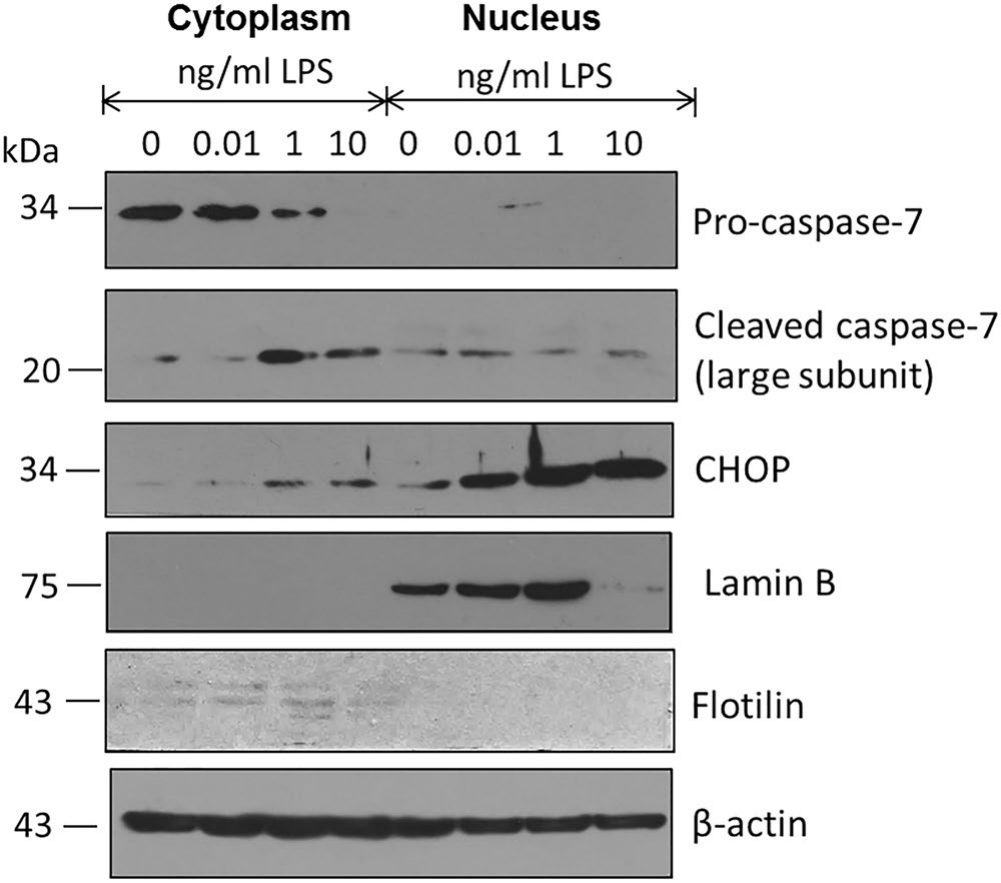
LPS treatment promotes cleavage activation of caspase-7 in the cytoplasmic fraction of macrophages. While the presence of pro-caspase-7 in the cytoplasmic fraction decreases with high LPS concentrations in the culture media of macrophages, the presence of caspase-7 increases in parallel with an important increase of CHOP in the nuclear fraction. Lamin-B used as a control associated to the nuclear fraction, flotilin for the cytoplasmic one and β-actin as the general loading control.

Caspase-7 or apoptosis-related cysteine peptidase 7 (CASP7) as a member of the caspases family, plays a central role in the execution-phase of cell apoptosis when activated through the proteolytic cleavage of the non-mature protein procaspase-7^21,22^. Interestingly, Fig. 3 shows that while procaspase-7 decreases in the cytoplasmic fraction in response to increasing concentrations of LPS in the cell culture media, the presence of the mature form CASP7 increases. Also, the CCAAT-enhancer-binding homologous protein (CHOP) as a multifunctional transcription factor associated to endoplasmic reticulum stress responses and inductor of cytokine production in macrophages^23,24^, dramatically increases its concentration mainly in the nuclear fraction, again in response to increasing concentrations of LPS (Fig. 3).

**Figure 4:**
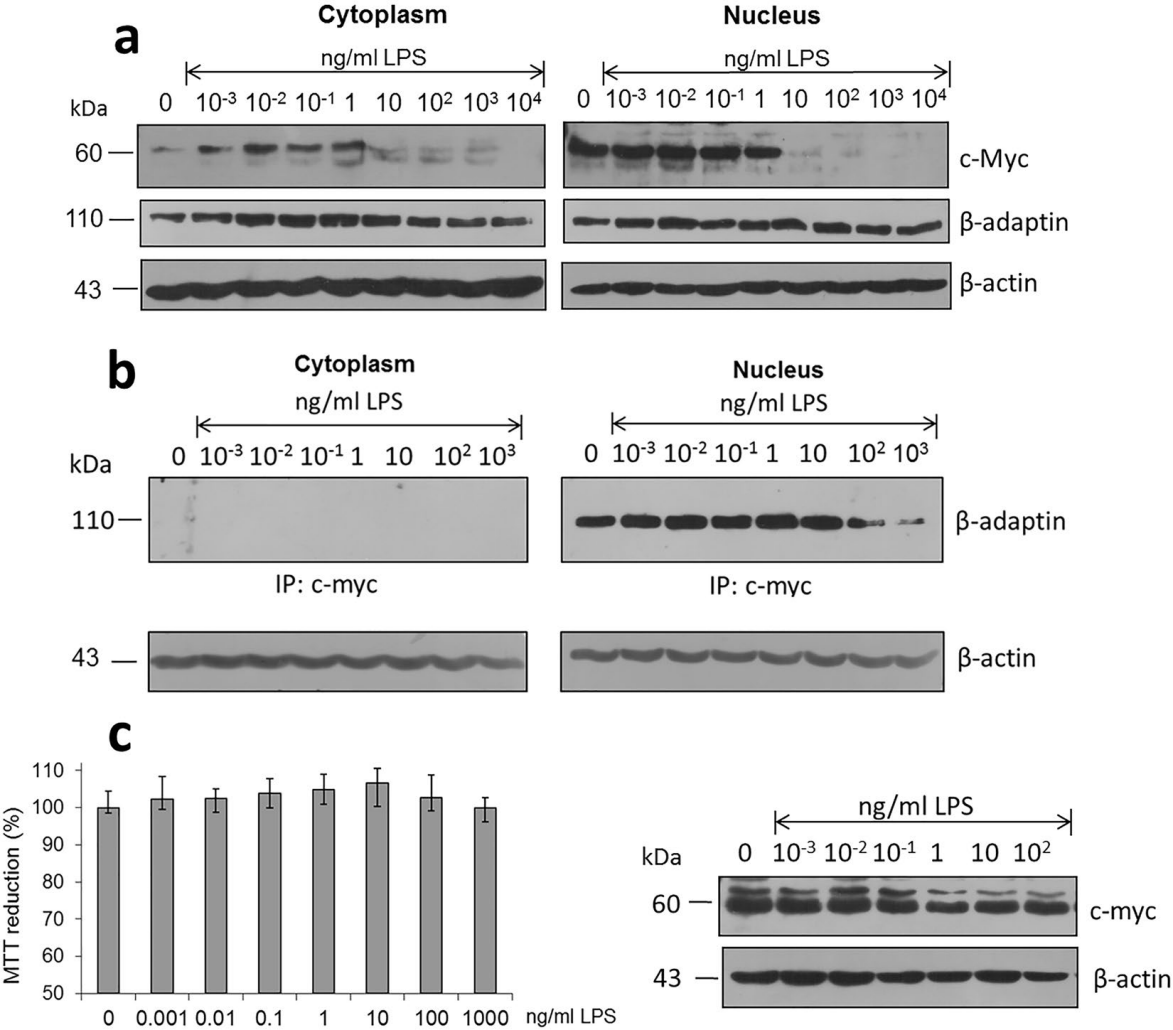
LPS treatment modifies the interaction between c-Myc and β-adaptin in the nucleus. (**a**) Expression levels of c-Myc and β-adaptin in cell extracts isolated from cytoplasm and nucleus under increasing concentrations of LPS. (**b**) Characterization of the interaction among β-adaptin and c-Myc under LPS treatment. (**c**) LPS treatment does not trigger hepatocyte cytotoxicity phenomena. Cell viability assays during a 20 h of treatment, and western blot detection of c-myc (**d**). β-actin used as a control. Mean values are presented (n = 6, X ± SD).

Next, we evaluated the expression of endocytic proteins in relationship with those directly involved in the regulation of cell cycle such as the proto-oncogen c-Myc (Fig. 4). Incubation with LPS 10ng/ml representing the boundary between the generation of an oxidative stress condition and a cell viability decrease, matches with a reduction in the concentration of cytoplasmic and nuclear c-Myc, possibly as a mechanism to initiate cell arrest in order to counterbalance inflammatory conditions and ameliorate foam cell formation.

While levels of β-adaptin in the nuclear and cytoplasm fractions remained constant throughout the highest LPS concentration tested (10^4^ ng/ml), the expression of c-Myc is suppressed with LPS concentrations starting at 1 ng/ml (Fig. 4a). In order to evaluate the possibility that β-adaptin could directly interact with c-Myc as a master regulator, immuno-precipitation assays were carried out employing both the nuclear and the cytoplasmic fractions. Under our experimental conditions, c-Myc was only immuno-precipitated in the nuclear fraction, situation that determines its capacity to interact with β-adaptin, condition found to be suppressed when cells are treated with high LPS concentrations (100 ng/ml) in accordance to results showing that synthesis of c-Myc is compromised under a state of oxidative stress (Fig. 4a,b).

In contrast, when similar experiments using the LPS stimulus are performed in liver-derived cells (HepG2) as control experiments for a macrophage specific response, a reduction in the percentage of cell viability is not registered, ROS levels remain constant at baseline levels with respect to controls (Fig. 2c) and expression levels of c-Myc maintained without significant changes (Fig. 2d). When cell viability under increasing concentrations of LPS is evaluated in a different hepatocyte cell line (C9 cells), the same response is registered (data not shown). Since there are no cell viability changes and the drop in c-Myc expression is not registered under an oxidative stress situation when hepatocytes are employed, the decrease in c-Myc expression associated to an important viability change seems to be associated to a macrophage specific response.

Since macrophages present the ability to change their functional phenotype depending on the environment they are exposed to, differentiation from M1 to M2 and vice versa has been considered^25,26^. In our hands, we have constantly observed that exposure of macrophages to an adverse environmental condition, not only employing LPS but also oxLDL particles, always shows an impact not only in cell viability but also in ROS production (Supplementary Fig. 1), favoring the M1 phenotype. Therefore, under this type of condition using oxLDL and considering the role these chemically modified lipoprotein particles play in the development of atherosclerosis, we next evaluated the expression of c-Jun and c-Myc when cells are exposed to oxLDL.

**Figure 5:**
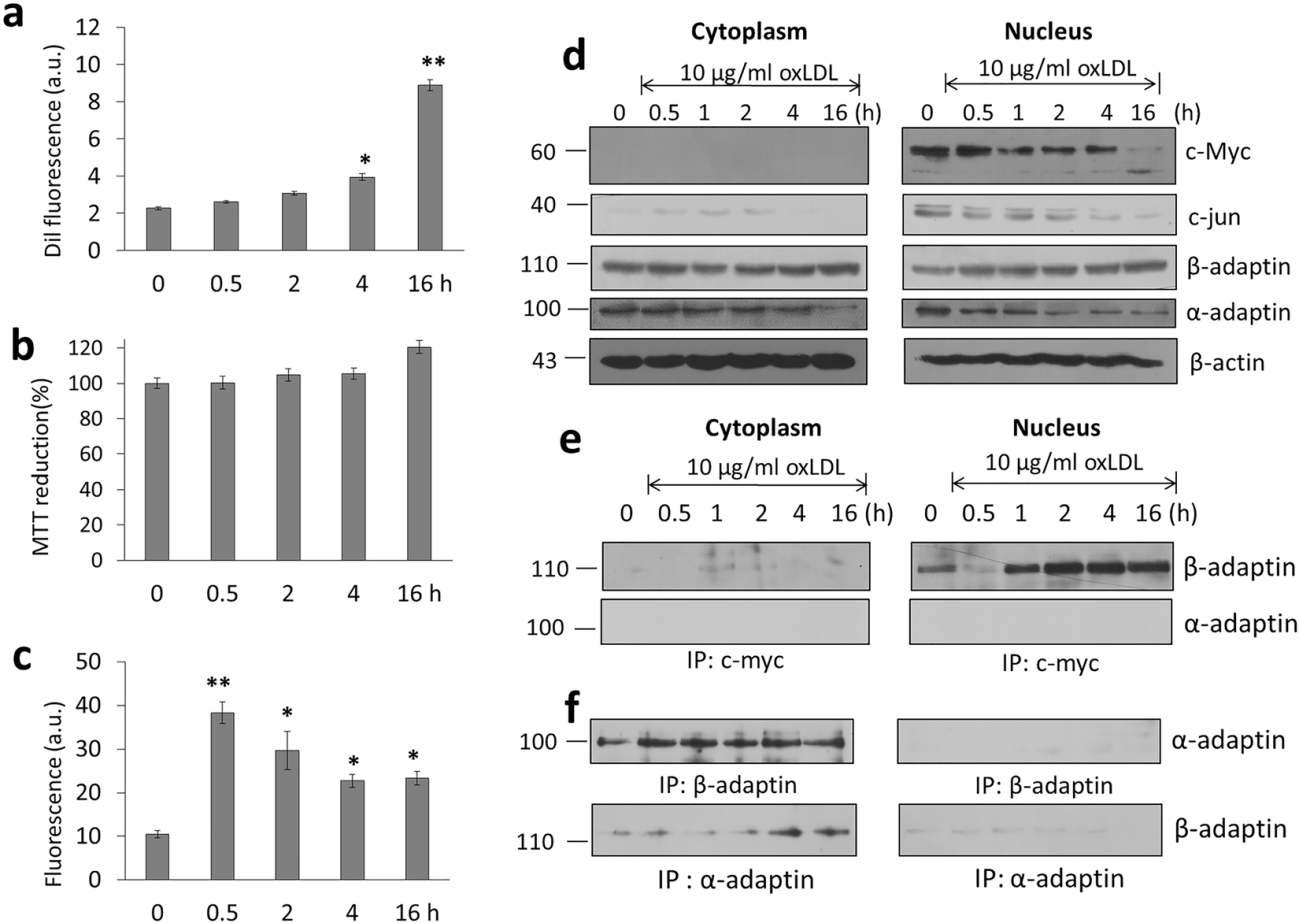
Internalization of oxLDL induces changes associated with the role of c-Myc and β-adaptin. (**a**) Internalization of oxLDL(10 µg/ml) in macrophages during an interval of 0–16 h. (**b**) Under the same experimental conditions, cell viability was evaluated. (**c**) Fluorescence associated to ROS in macrophages treated with oxLDL. (**d**) Protein expression profile of β-adaptin, α-adaptin, c-Jun and c-Myc in nucleus and cytoplasm isolated. (**e**) Western blot of immunoprecipitation experimentation of c-Myc in macrophages incubated with oxLDL, detection of β-adaptin. (**f**) Control experiment for the interaction α-adaptin/β-adaptin, part of the complex AP2. In all cases β-adaptin used as control. Mean values are presented (n = 6, X ± SD) *p < 0.05, **p < 0.01.

Employing 10 µg/ml oxLDL and incubation time intervals between 0 and 16 h, the internalization of oxLDL was evaluated (Fig. 5a). Although this condition does not alter cell viability during treatment for 16 h (Fig. 5b), an increase in ROS generation is observed recording the highest concentration point at the start of the experiment with a tendency to be stabilized after 16 h incubation (Fig. 5c), conditions considered as a moderate stress while still maintaining cell basic functions. Under these conditions, when c-Myc and c-Jun are evaluated, similar to results obtained with LPS treatment, a decrease in their expression and presence in the nucleus is registered (Fig. 5d). Moreover, while oxLDL treatment causes a slight drop in the expression of adaptor protein α-adaptin, β-adaptin maintains its concentration in both the nuclear and cytoplasmic fractions. Although along with time, the nuclear localization of c-Myc tends to decrease, when immuno-precipitation experiments are carried out, it is evident there is an increased interaction between β-adaptin and c-Myc when cell are exposed to oxLDL (Fig. 5e). The interaction between α-and β-adaptin, as part of the endocytic complex adaptor AP2 was used as a control (Fig. 5f).

**Figure 6:**
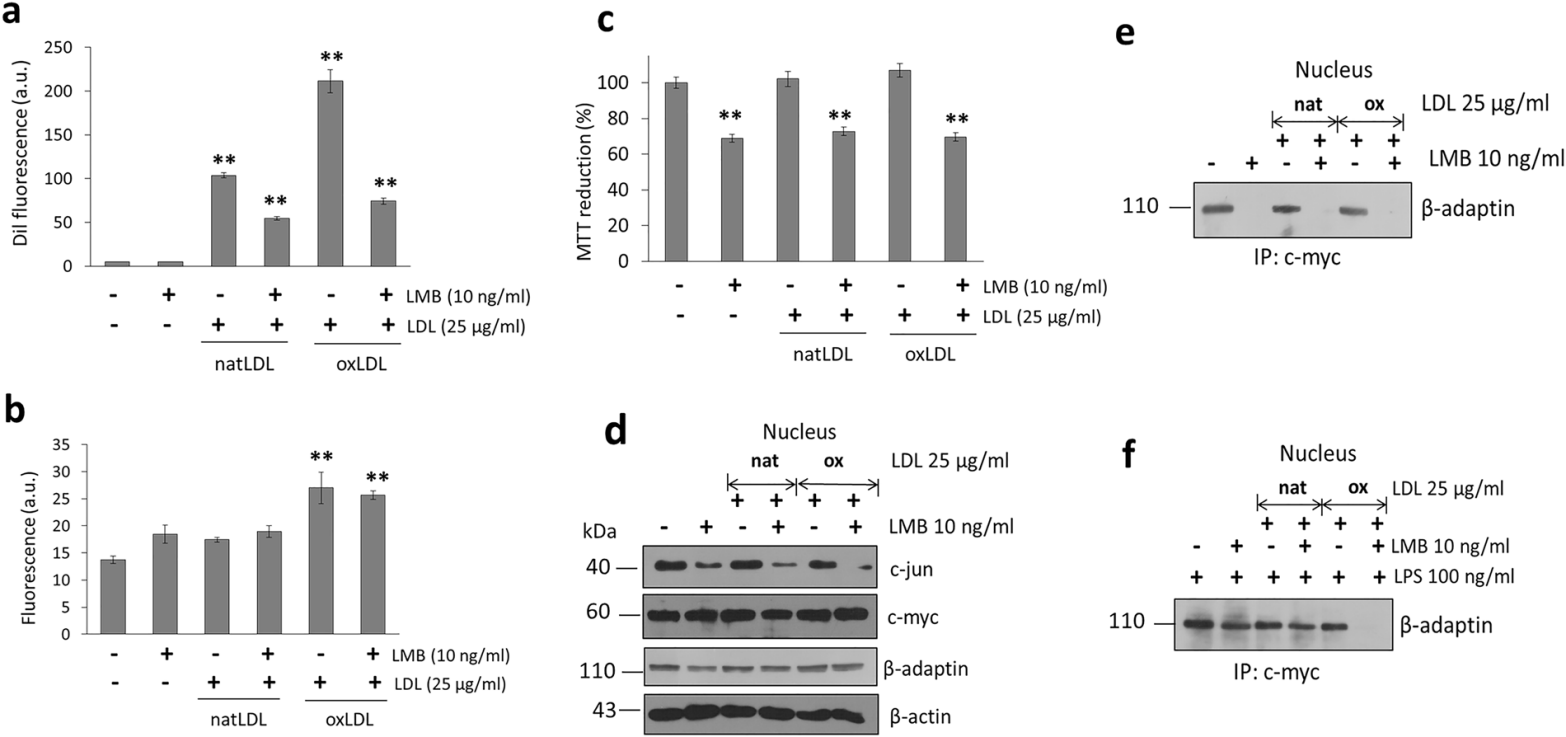
Inhibition of nuclear-cytoplasmic transport modifies the internalization of oxLDL, associated with disruption of the interaction c-Myc and β-adaptin. (**a**) Effect of LMB treatment (4 h) on internalization of dil-fluorescent natLDL and oxLDL in macrophages. (**b**) Under the same conditions, ROS characterization was performed. (**c**) Evaluation of cell viability by the MTT assay. (**d**) Western blot detection of c-Jun, c-Myc and β-adaptin under the treatment with LMB, natLDL and oxLDL. (**e**) Characterization of β-adaptin and c-Myc interaction. (**f**) Effect of LPS stimulus (100 ng/ml) on the interaction c-Myc and β-adaptin, and pretreatment with α-tocopherol (300 µM). Mean values are presented (n = 6, X ± SD) *p < 0.05, **p < 0.01.

Taking into account the distribution in the cytoplasm and the nucleus of several proteins that participate in the process of endocytosis and their potential new role during an oxidative stress conditions, we carried out the inhibition of the chromosomal region maintenance protein (CRM1) employing leptomycin B (LMB). This molecule is known to cause cell cycle arrest in G1 and reported to inhibit the nuclear export of many proteins and translation of several RNAs including COX-2 and c-Fos^27^. Treatment of macrophages with a dose of 10 ng/ml LMB (4 h) induced a decrease in the process of internalization of both natLDL and importantly of oxLDL (Fig. 6a), with a close association to ROS generation (Fig. 6b) and a reduction in cell viability (Fig. 6c).

Employing the same conditions, the nuclear distribution of c-Jun and c-Myc under LMB treatment in the presence of natLDL or oxLDL was studied. While c-Myc tends to maintain the same concentration regardless of treatment, c-Jun concentration decreases with LMB (Fig. 6d). The decrease in the levels of c-Jun is almost complete when cells treated with LMB are kept in the presence of oxLDL (Fig. 6d). As observed before, levels of nuclear β-adaptin are not at all changed (Fig. 6d). Although c-Myc present in the nucleus seems not to be affected by LMB, immuno-precipitation experiments show that under these experimental conditions, cells do not show c-Myc/β-adaptin interactions (Fig. 6e). Since c-Myc is apparently retained in the nucleus and its interaction with β-adaptin altered, this result explains an effect upon the way cell cycle works, phenomenon that is reflected on the fact that LMB also interferes with cell viability (Fig. 6c). Interestingly, during the same set of experiments, it is observed that the presence of oxLDL does not change cell viability percentages observed with LMB treatment only (Fig. 6c). Altogether, these results show that LMB treatment directly influences the nuclear localization of c-Jun and modifies c-Myc/β-adaptin interactions. When cells are preincubated in the presence of the antioxidant molecule α-tocopherol and the same kind of experiment performed, this condition restores the nuclear interaction between c-Myc and β-adaptin (Fig. 6f). Interestingly, α-tocopherol pretreatment while interfering with the internalization process of both natLDL and oxLDL, seems to protect cells from producing a critical ROS concentration allowing cells to maintain cellular homeostasis (Supplementary Fig. 2). Therefore, in general it seems treatment with α-tocopherol improves macrophage homeostasis maintaining the normal development of the cell cycle by the formation of the newly described β-adaptin/c-Myc complex. Although the use of α-tocopherol has been shown to be effective against the action of oxLDL during proliferation in smooth muscle cells^28^, the new role for novel regulatory complexes such as the one described here formed between β-adaptin and c-Myc, has not been pointed out before.

**Figure 7:**
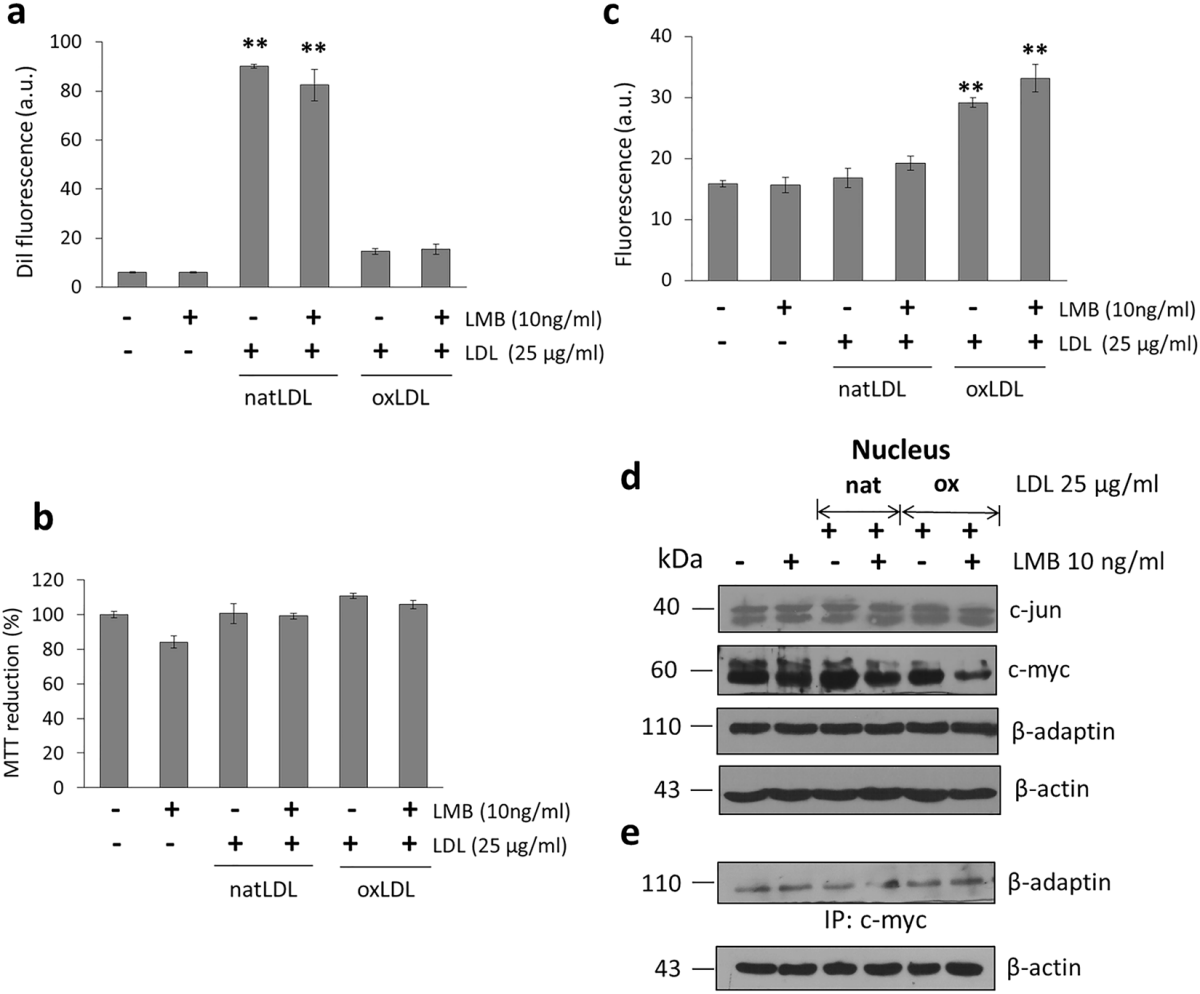
Short LMB treatment of hepG2 cells in culture while internalizing natLDL and oxLDL. (**a**) Response to a LMB treatment upon internalization of natLDL and oxLDL (Dil) in hepG2 cells. (**b**) Cell viability measured by MTT assays. (**c**) ROS production by LMB treated hepG2 cells while in the presence of natLDL and oxLDL. (**d**) Western-blot analysis of proteins c-Jun, c-Myc, and β-adaptin present in hepG2 cells while treated with LMB in the presence of natLDL or oxLDL (**e**) Immunoprecipitation assay to evaluate the interaction between c-Myc and β-adaptin in hepG2 cells treated with LMB in the presence of natLDL or oxLDL. Mean values are presented (n = 6, X ± SD) *p < 0.05, **p < 0.01.

On the other hand, hepG2 cells derived from the embryonic endoderm in comparison to macrophages that present a mesodermal origin, when stimulated with natLDL and oxLDL, only internalization of natLDL is recorded (Fig. 7a). Independently of these results, it is interesting to observe that although no internalization of oxLDL is achieved, a parallel modest increase in the production of ROS is recorded while cell viability seems not to be affected (Fig. 7b,c). Under these experimental conditions, in comparison to results obtained with macrophages, HepG2 LMB treated cells do not present an effect, the expression of c-Jun is not affected and only a slight decrease in the expression of the proto-oncogene c-Myc registered (Fig. 7d). It is interesting to observe that particularly with cells under treatment with oxLDL, the interaction β-adaptin/c-Myc is not modified by LMB (Fig. 7e), in contrast to the response observed with macrophages.

When HepG2 cells are further subjected to an additional LPS stimulation, a slight exacerbation of results previously obtained are observed. A slight increase in the internalization of natLDL is recorded, an effect that seems to be reduced under LMB treatment (Supplementary Fig. 3a). In the same fashion as previously shown, LPS incubation does not alter the fact that oxLDL are not internalized by these cells but associated to an increase in ROS concentration (Supplementary Fig. 3b). The same type of response is also registered when HEK293 cells were evaluated (data not shown).

In this sense, experiments performed with HepG2 cells and macrophages show that incubation in the presence of oxLDL, lead to a time dependent increased expression of SR-B1 (0–16 h) (Supplementary Fig. 4a,b). This increased expression of SR-BI in hepatocytes might correspond to a cellular response designed to counteract cytotoxic signaling pathways that could lead to the synthesis of pro-inflammatory molecules. Moreover, as previously shown by us, the interaction of β-adaptin with SR-BI during a state of cellular oxidative stress supports the role this protein plays during several adaptive functions initiated by abnormal cell growing conditions^10^. Now, we further support the fact that β-adaptin, under abnormal conditions such as an oxidative stress state, disassociates from c-Myc and the complex disrupted, modifying the progression of the cell cycle. Unlike the association of β-adaptin with c-Myc, the interaction between β-adaptin and SR-BI is not modified by incubation with LMB (data not showed).

**Figure 8:**
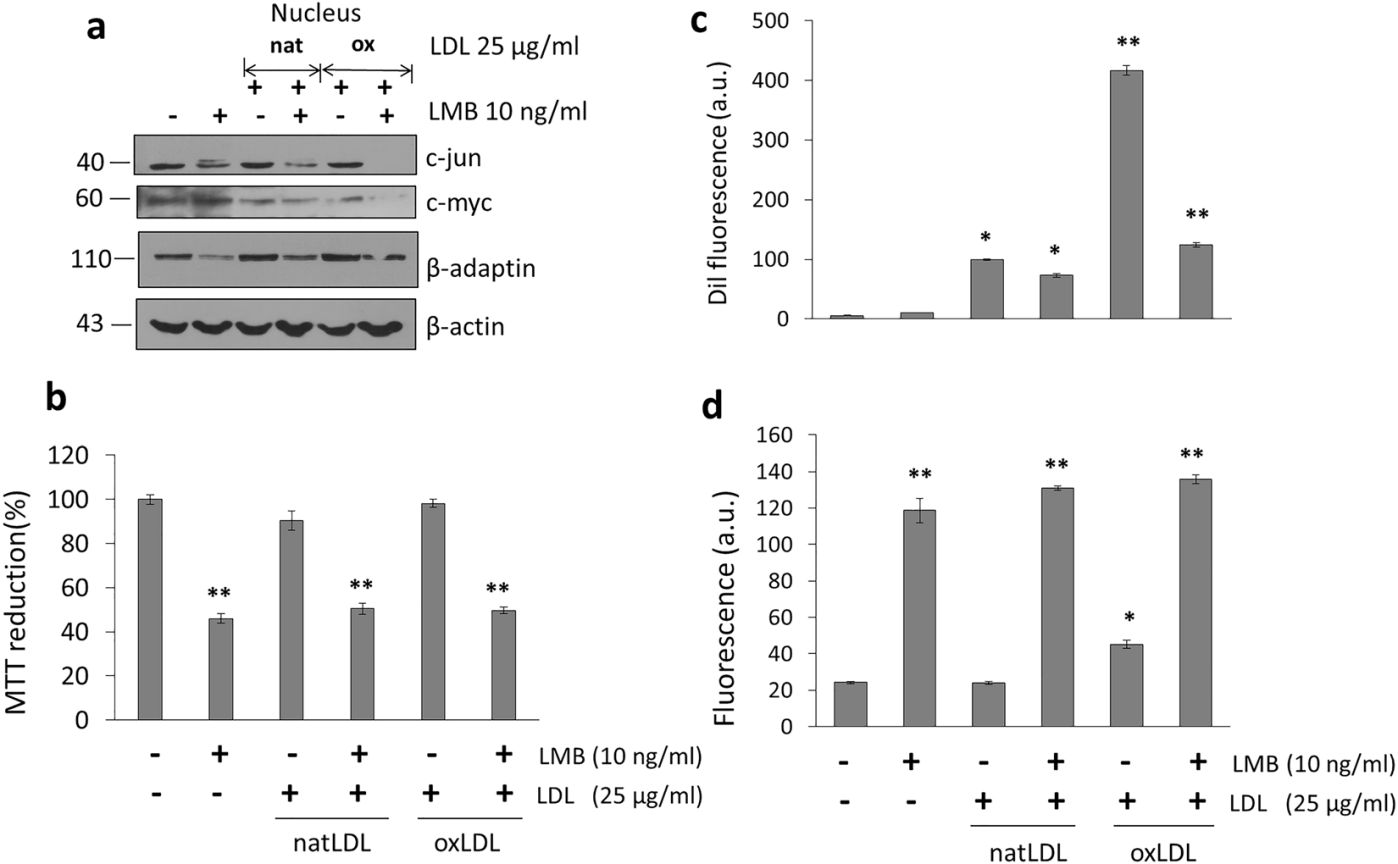
A prolonged LMB treatment (16 h) of macrophages induces a state of severe oxidative stress. (**a**) Western blot analysis in the expression of proteins c-Jun, c-Myc and β-adaptin present in the nuclear fraction after a prolonged LMB treatment in the presence of natLDL and oxLDL. (**b**) Under the same conditions, cell viability obtained by MTT assays. (**c**) LMB effect upon internalization of dil-fluorescently labeled natLDL and oxLDL. (**d**) ROS characterization under the same conditions. Mean values are presented (n = 6, X ± SD) *p < 0.05, **p < 0.01.

**Figure 9:**
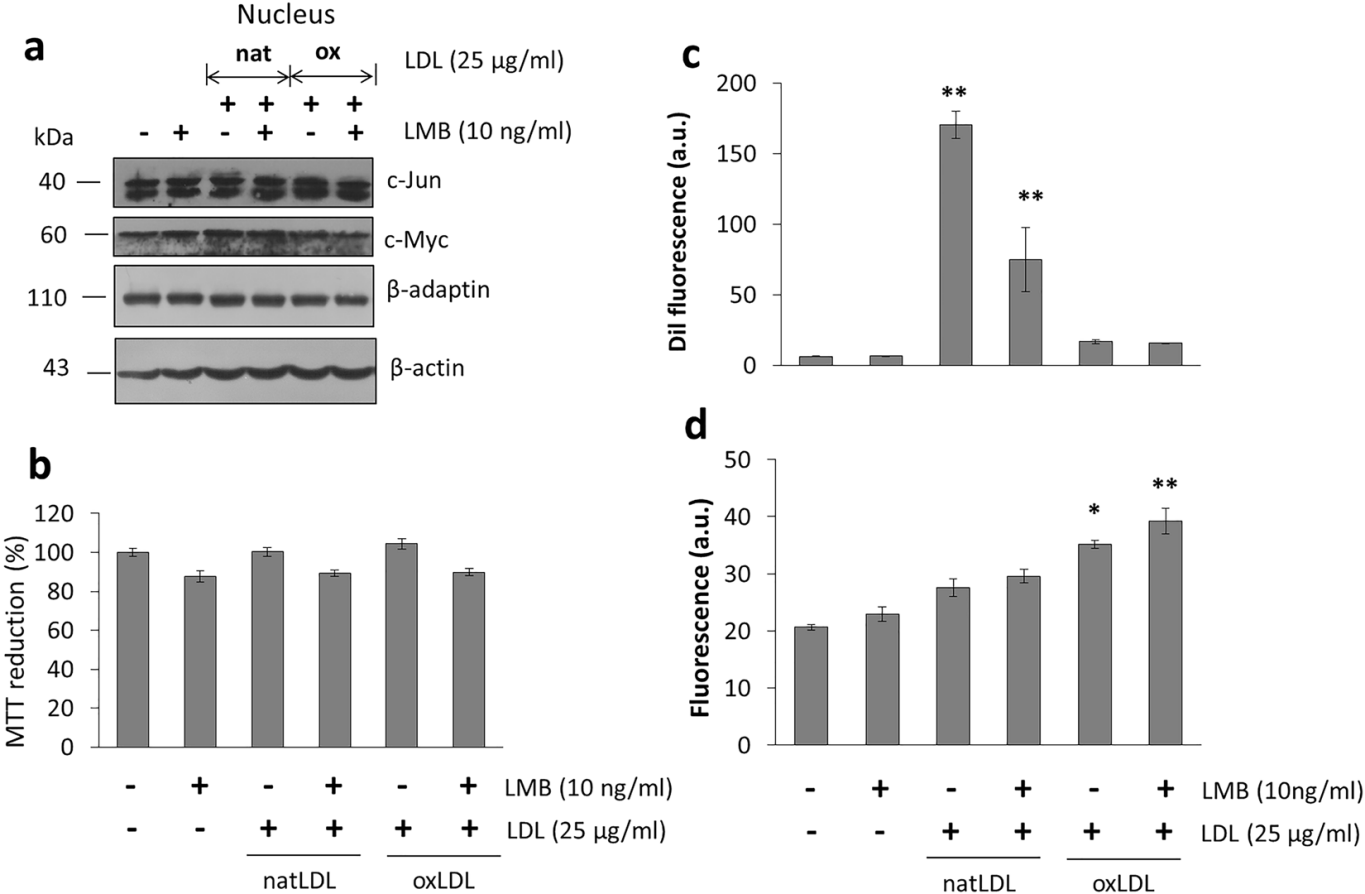
A prolonged LMB treatment (16 h) in hepG2 cells induces a state of severe oxidative stress. (**a**) Western blot analysis in the expression of proteins c-Jun, c-Myc and β-adaptin present in the nuclear fraction after a prolonged LMB treatment in the presence of natLDL and oxLDL. (**b**) Under the same conditions, cell viability obtained by MTT assays. (**c**) LMB effect upon internalization of dil-fluorescently labeled natLDL and oxLDL. (**d**) ROS characterization under the same conditions. Mean values are presented (n = 6, X ± SD) *p < 0.05, **p < 0.01.

Although after 4 h incubation in the presence of LMB there is an accumulation of proteins in the nucleus, a chronic exposure for 16 h, seems to extensively affect mRNA transport in macrophages. This situation affects the concentration of proteins such as β-adaptin, c-Jun and c-Myc, associated with the presence of high levels of ROS, a sharp fall in cell viability and a decreased internalization of oxLDL (Fig. 8). Several authors have indicated that LMB treatment induces the modification of cellular mechanisms such as autophagy through the interference in the transport of key proteins^29^, and in some cases promoting the formation of protein aggregates^30^. In the case of hepatocytes, if stimulation with LMB is prolonged for up to 16 h, only a drastic drop in the internalization of natLDL particles (56%) is recorded without any significant increases in ROS production (Fig. 9), therefore altering only the endocytic activity. From the physiological point of view, an alteration in the internalization process of natLDL by hepatocytes, could be associated with a longer circulation time of these particles in the plasma, presenting a higher possibility to develop a chemical modification and therefore exacerbating the possibility to participate in the induction of atherogenesis.

## Discussion

Our results showing that the establishment of a state of oxidative stress in the macrophage seem to profoundly affect the expression of transcriptional activators such as c-Myc and c-Jun, the expression of cytoskeletal protein β-adaptin and the formation of a β-adaptin/c-Myc complex in the nucleus becomes a key point in the understanding of cell survival under adverse conditions. The formation of such a complex with reversible characteristics has allowed us to describe a new property for β-adaptin during the establishment of a state of oxidative stress regulating the activity of c-Myc and therefore the development of the cell cycle.

In this respect, ongoing work from our laboratory investigating the process of endocytosis has shown that during an oxidative stress condition, adaptor proteins such as BIN1 also regulate c-Myc function^13^. The formation of a BIN1/c-Myc heterodimer detected when oxLDL is being internalized, suggests that through this mechanism, cells respond to a stressful condition by inhibiting the progression of the cell cycle and therefore providing cells with a potential mechanism for survival^13^. Several reports have demonstrated that oxLDL might influence the activation and expression levels for c-Myc in the atherosclerotic plaque^28^. Although, tocopherol was shown to improve the action of oxLDL upon c-Myc^28^, the new regulatory role exerted by β-adaptin described here has not being shown before. Therefore the kind of interaction carried out between c-Myc and β-adaptin with the capacity to modulate the cell cycle in the macrophage, may be considered a cell specific response to cell damage in the regulation of foam cell formation as an initial step in the process of atherogenesis.

The differential results obtained with macrophages and hepatocytes suggest the presence of cell specific response patterns developed following an adverse environmental situation in association to mechanisms that regulate the nuclear localization of transcription factors such as c-Jun and c-Myc. This novel finding that takes place with the reversible formation of a β-adaptin/c-Myc complex, also supports the fact that this type of association influences the nucleus-cytoplasm transport of proteins. Our present finding is supported by previous experimental data from our laboratory showing that a state of oxidative stress, while maintaining cell viability around 50% caused by the presence of peptide β-amyloid in the culture media of macrophages and microglial cells, promotes a decrease in the concentration of cytoplasmic β-adaptin in parallel with an increase in the concentration of scavenger receptor SR-B1^10,11^. Since we now know that due to the presence of a high ROS concentration and a state of severe oxidative stress, complexation between c-Myc and cytoskeletal proteins like β-adaptin is affected, our results support the importance shown by the nucleus-cytoplasm transport of proteins in the control of the cell cycle.

Although reactive oxygen and nitrogen species for years now have been known to play a key role during the development of several important cell functions, nowadays it is clear that calcium also plays an important role in cell death disrupting normal physiological pathways by triggering either necrotic or apoptotic cell death. Since a state of oxidative stress causes a calcium influx into the cytoplasm either from internal stores such as the endoplasmic reticulum and the mitochondria or from the extracellular space, a concentration increase of calcium in the cytoplasm has been linked with many diseases including atherosclerosis^31–33^. Our results showing an important correlation between the response macrophages show when treated with high concentrations of LPS and an increase in the concentration of PMCA in the cell nucleus and cleavage activation of procaspase-7 in the cell cytoplasm, indicate that the state of oxidative stress achieved in our macrophage model is probably also associated to a calcium unbalance. Although throughout our study we have used a macrophage cell line and new experiments are needed employing human purified primary monocyte/macrophages, taken together, the phenomena described in our study correlating the generation of a state of oxidative stress and cell survival, allow us to propose that cells by adapting to a new stressful condition acquire the ability to control the development of the cell cycle by adjusting the formation of complexes between nuclear and cytoplasmic proteins in parallel to the over-expression of proteins with a protective action against the damaging effect of high intracellular concentrations of calcium.

The discovery of a β-adaptin/c-Myc complex capable to modulate the cell cycle in macrophages under conditions of oxidative stress by exposure to LPS or pro-atherogenic oxLDL particles, together with a compromised functional endocytic capacity and the parallel loss of cell calcium homeostasis, may be considered a relevant process in the understanding of how macrophages under adverse conditions are able to maintain a normal cell homeostasis.

## Materials and Methods

### Materials

All cell culture reagents were purchased from Gibco-Invitrogen (Carlsbard, CA, USA). Cell culture dishes and other plasticware were obtained from Nalgene Nunc (Rochester, NY, USA). Salts and buffers were purchased Sigma (St. Louis, MO, USA). Leptomycin B (LMB), α-tocopherol, LPS O111:B4 and (3-(4,5-dimethylthiazol-2-yl)-2,5-diphenyltetrazolium bromide) tetrazolium (MTT) were obtained from Sigma-Aldrich. Primary antibodies used in protein detection of CALM, α-adaptin, β-adaptin, eps 15, c-Myc, c-Jun, c-Abl and SR-B1 as well as secondary antibodies were from Santa Cruz Biotechnology (Santa Cruz, CA, USA) and Abcam (Cambridge, UK). Fluorescent conjugates of LPS BODIPY FL, and 6-carboxy-2′,7′-dichlorodihydrofluorescein diacetate for ROS detection were obtained from Thermo Fisher Scientific (Waltham, MA, USA).

### Cell Culture

The macrophage/monocyte RAW 264.7 cell line derived from virus induced rat macrophages (ATCC) was grown in RPMI 1640 medium using 10% fetal bovine serum (FBS). Human derived epithelial liver HepG2 cells (ATCC), C9 derived rat epithelial liver cells (ATCC) and HEK293 derived from human embrionary epithelial kidney cells (ATCC) were grown in DMEM using 10% FBS and sodium pyruvate (1 mM). Penicillin (50 U/ml) and streptomycin (50 μg/ml) were added to the media.

### Statistical analysis

Data are expressed as mean ± SD. Comparisons between groups were made using Student’s t test.

### Cell Viability Assay

Cytotoxicity was assessed by MTT reduction assays in RAW, HepG2, C9 and HEK293 cells exposed to different stimuli. Cells were seeded into 96-well plates at a density of 14000 cells/well and allowed to grow to 90% confluence. Next, proliferation culture medium was replaced with Opti-MEM. After 2 h under this condition, cells were treated under several specific conditions. Later, 30 μl of a MTT stock solution in Opti-MEM medium was added to the culture media in order to obtain a final concentration of 0.5 mg/ml. Formazan crystals that formed after 4 h of incubation were further dissolved by the addition of a buffer that causes lysis (20% SDS, 50% N,N-dimethyl-formamide, pH 3.7). After 12 h incubation, absorbance was measured at 570 nm using a microplate reader^11^.

Cell cultures placed in 96 well plates (14000 cells/well) at 90% confluence were incubated with different concentrations of LPS, LMB and LDLs. After treatment, cell viability was measured through the MTT assay. In a different set of experiments, macrophages received 10 ng/ml LMB stimuli (10 ng/ml) for 4 and 16 h, prior the addition of a gradually increasing concentration of natLDL or oxLDL. These experiments were prolonged for additional 16 h and MTT assays performed according to the protocol previously used^11,34^.

### Reactive oxygen species characterization

Macrophages placed on black 96-well plates were incubated for 1 h in serum-free medium, before being treated with LMB (10 ng/ml) for 4 or 16 h. LDL particles were added and cells maintained in this medium for 16 h at 37 °C. At the end of this period, the fluorescent probe 6-carboxy-2′,7′-dichlorodihydrofluorescein-diacetate (11 μM) was added to the culture medium followed by further incubation for 45 min. Cells were washed and fluorescence measured in a Biotek Synergy-HT microplate reader.

### Western blot analysis and immunoprecipitation assays

Employing a 90% confluence, cells were treated with the different stimulus studied. Afterwards, cells were washed with PBS and lysed for 45 min at 4 °C in lysis buffer. Lysed cells were centrifuged at 9000 rpm for 10 min and the supernatant recovered. Protein concentration was determined with the BCA protein assay (Pierce, Rockford IL, USA) and samples (20 μg/lane) from the total protein fraction analyzed by SDS-PAGE on 8% gels and further transferred to PVDF membranes (Millipore). Membranes were blocked overnight at 4 °C in a solution containing TBS, 0.1% tween-20, and 5% non-fat milk. For protein detection, the following primary antibodies were used: anti-β-adaptin, anti-α-adaptin, anti-CALM, anti-eps 15, anti c-Jun, anti c-Myc, anti-PMCA, anti-CD47, anti-pro-caspase-7 and anti-c-Abl. In addition, we used anti β-actin (1:500). Blocked membranes were incubated with the primary antibodies for 1 h at 37 °C. After washing, horseradish peroxidase (HRP) conjugated secondary antibodies (1:5000) were incubated with the membrane for 1 h at 37 °C in blocking solution. The secondary antibodies used were: donkey anti-goat IgG, goat anti-rabbit IgG, and goat anti-mouse IgG. Later, membranes were washed with TBS/0.1% tween and HRP activity detected with the Immobilon Western kit (Millipore).

Nuclei separation was carried out using a buffer containing sucrose (250 mM) and imidazole (3 mM) pH 7.4, supplemented with protease and phosphatase inhibitors. Cells were scraped from culture dishes and 21 passages were performed through a 22 G syringe. For the recovery of nuclei, lysates were centrifuged at 3400 rpm for 15 min. The two fractions (supernatant and pellet) were lysed for 25 min at 4 °C, and both fractions (20 μg/lane) were analyzed by SDS-PAGE and transferred to PVDF membranes. Membranes were incubated with primary antibodies and after successive washes, membranes were incubated with their respective secondary antibodies (1:5000) and horseradish peroxidase activity detected. Controls for the nuclear and cytoplasmic separation were performed by identification of anti-Lamin-B, anti-flotillin and anti-GAPDH.

For the immuno-precipitation assays, cytoplasmic (400 μg) and nuclear (300 μg) fractions were incubated with an anti-c-Myc antibody (1:400) for 2 h at 4 °C. Immune complexes were precipitated with protein G agarose Fast Flow (Millipore) 12 h at 4 °C. Immuno-precipitated proteins were washed 3 times and suspended in Laemmli buffer, separated by SDS-PAGE gels and transferred to PVDF membranes for western-blot analysis. Protein detection was performed based on previous described protocols.

### LDL fraction

Human plasma samples were obtained from healthy donors at the National Medical Center “20 de Noviembre”, Mexico, that signed an informed consent. All protocols were performed according to the Declaration of Helsinki. To isolate LDL, plasma density was adjusted to a 1.019–1.063 g/ml by adding KBr and then centrifuged at 360,000 g for 8 h at 4 °C. The layer containing VLDL and IDL was discarded. LDL preparation was recovered and dialyzed against at 150 mM NaCl, filtered through 0.45 μm filters and stored under a nitrogen atmosphere at 4 °C to reduce oxidation. Native LDL (natLDL) concentration was measured with the bicinchoninic acid method (BCA).

To prepare oxLDL, natLDL (100 µg/ml) was incubated with a 10 μM CuSO_4_ solution in PBS without calcium, magnesium nor antioxidants at 37 °C for 4 h. The reaction was stopped with 100 μM EDTA and the preparation dialyzed against 150 mMNaCl containing 240 μM EDTA and stored at 4 °C until use. oxLDL concentration was estimated by reading in the UV range (218 nm) using a UV/VIS Lambda 2 S (Perkin-Elmer) spectrophotometer.

Oxidation was terminated with the addition of EDTA, and the resulting oxidized LDLs (oxLDLs) were dialysed against 150 mM NaCl containing 100 μM EDTA and then stored at 4 °C. Labeling of LDL with 1,1′-dioctadecyl-3,3,3′3′-tetramethylindocarbocyanine-perchlorate (DiI) (Molecular Probes) was performed as previously described^35,36^. Isolation of the HDL fraction from human plasma (density 1.063–1.21 gKBr/ml) was performed according to established methods and based on a protocol described by Toledo-Ibelles *et al*.^37^ Samples were quantified by the BCA method.

For internalization assays, cells were incubated in the presence of Dil-labeled lipoproteins under different conditions employing 96-well plates containing 14000 cells/well. After the internalization assay, cells were washed and fluorescence read in a Synergy HT Microplate Reader (Biotek).

### LPS experimentation

LPS was dissolved in ultrapure water (1 mg/ml) and sonicated in a water bath for 10 min. LPS samples were added to cells according to the different protocols described.

### Cell cytometer assays

Prior to the internalization experiments, cell cultures at 90% confluence were incubated in FBS-free medium to synchronize the endocytic machinery. After a 1 h fasting condition, cells were treated with different LDL and LMB concentrations under a 5% CO2/95% air atmosphere. After the internalization studies were completed, cell cultures were washed with 2 mg/l albumin in PBS and fluorescence measured by flow cytometry using a FACSCalibur (BD Biosciences) equipment. In parallel, cell cultures used for internalization experiments were prepared for confocal microscopy or lysed for Western blot analysis.

### Confocal microscopy

A confocal scanning biological microscope FV1000 (Olympus) was employed in order to characterize the LDL and LMB effects. For these experiments, cells were incubated with different conditions employing LDL labeled with DiI for 4 h, washed with PBS and mounted for observation using excitation and emission wavelengths of 543 nm and 595 nm respectively. Additional treatments with LMB and α-tocopherol were also evaluated. ROS localization in cells was performed using the 6-carboxy-2′,7′-dichlorodihydrofluorescein-diacetate probe.

### Lipid peroxidation

Lipid peroxidation was estimated by the thiobarbiturate assay for thiobarbituric acid reactive substances (TBARS) based on the method originally described by Wilbur *et al*.^38^ and modified by us following a technique employed by Kovachich and Mishra^35,39^. Macrophages proliferating for 36 h were incubated for 12 h with LPS concentrations. After treatment, cells are resuspended in PBS, washed once and protein concentration measured. One ml of LPS treated macrophages adjusted to a protein concentration of 2 mg/ml are homogenized with 0.5 ml 40% trichloroacetic acid using a pestle homogenizer and centrifuged for two minutes at 2000 rpm. One ml supernatants are further mixed with 1.0 ml of 0.67% thiobarbituric acid and samples placed in boiling water for 10 min further centrifuged for two minutes al 2000 rpm. Supernatants are used for the measurement of optical density at 530 nm.

### Nitrites determination

Samples derived from the supernatant media and cytoplasmic fractions were processed for nitrite quantification employing the Griess reagent adapted by Giustarini^40^. A standard curve comprising 0.06–20 µM nitrites was constructed and measurements performed using a microplate reader Synergy HT (Biotek).

## Electronic supplementary material

Supplementary material
