# Supplementary material for: A Novel β-adaptin/c-Myc Complex Formation Modulated by Oxidative Stress in the Control of the Cell Cycle in Macrophages and its Implication in Atherogenesis
Source: Sci Rep. 2017 Oct 18;7:13442. doi: 10.1038/s41598-017-13880-5 (PMC5647411; doi:10.1038/s41598-017-13880-5)

A Novel β-adaptin/c-Myc Complex Formation Modulated by Oxidative Stress in the Control of the Cell Cycle in Macrophages and its Implication in Atherogenesis

Victor García-González & Jaime Mas-Oliva

Supplementary material

Supplementary fig. 1 | Treatment with increasing concentrations of oxLDL generates an oxidative state in macrophages. (**a**) Fluorescence values associated with the internalization of oxLDL labeled with DiI after 16 h. (**b**) Evaluation of ROS production under the same experimental conditions. (**c**) Cell viability through MTT assay. Confocal microscopy images of macrophages stimulated with 0 (**d**), 0.1 (**e**), 10 (**f**) and 100 μg/ml (**g**) oxLDL. Nucleus stained with Hoechst. Mean values are presented (n=5, X ± SD) $*p<0.05$, $**p<0.01$.

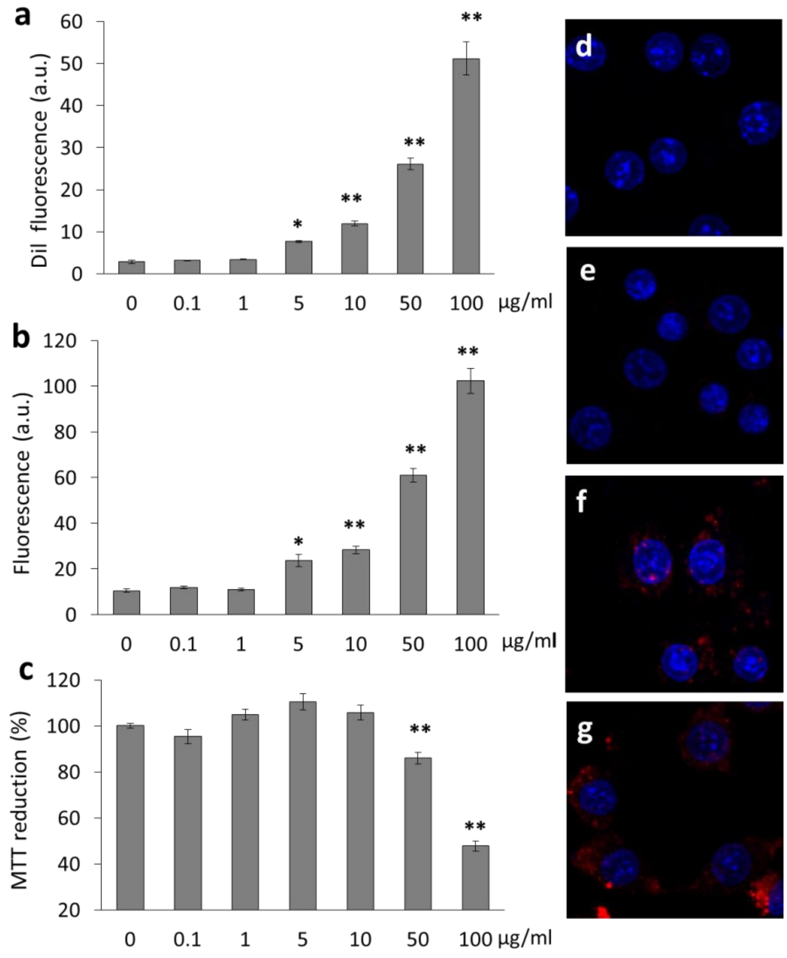

Supplementary fig. 2 | Effect of pretreatment with α-tocopherol in macrophages later stimulated with LMB, natLDL and oxLDL for 4 h. (**a**) Evaluation of particle internalization. (**b**) Characterization of ROS production. Mean values are presented (n=5, X ± SD) *p<0.05, **p<0.01.

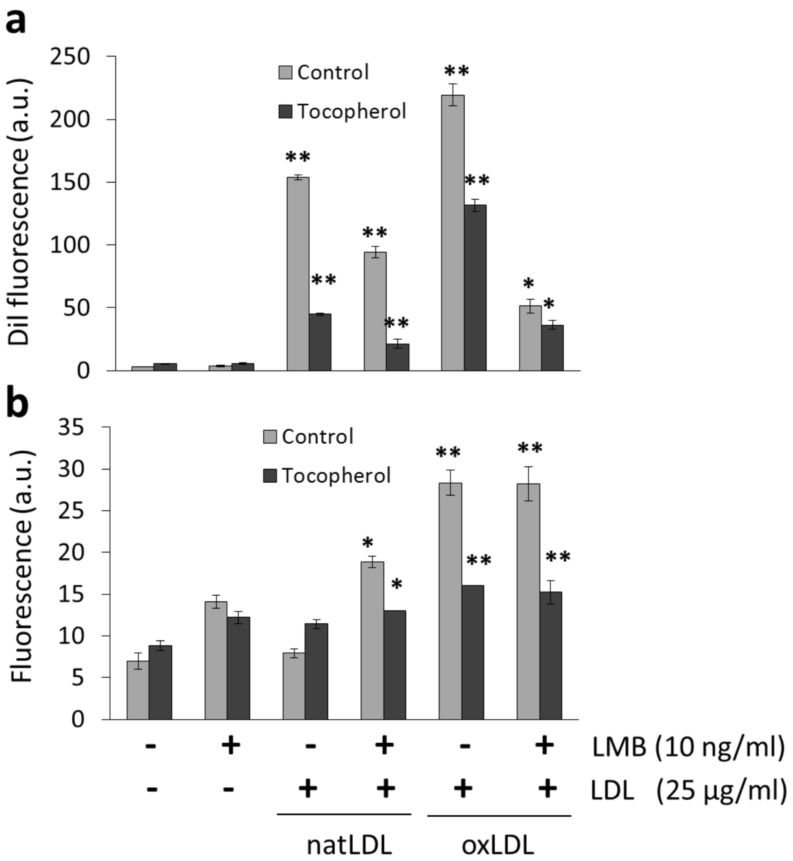

Supplementary fig. 3 | Effect of LPS treatment (100 ng/ml) in hepatocyte cultures stimulated with natLDL and oxLDL. **(a)** Evaluation of internalization. (**b**) Characterization of ROS production. . Mean values are presented (n=5, X ± S.E.M.), *p<0.05, **p<0.01.

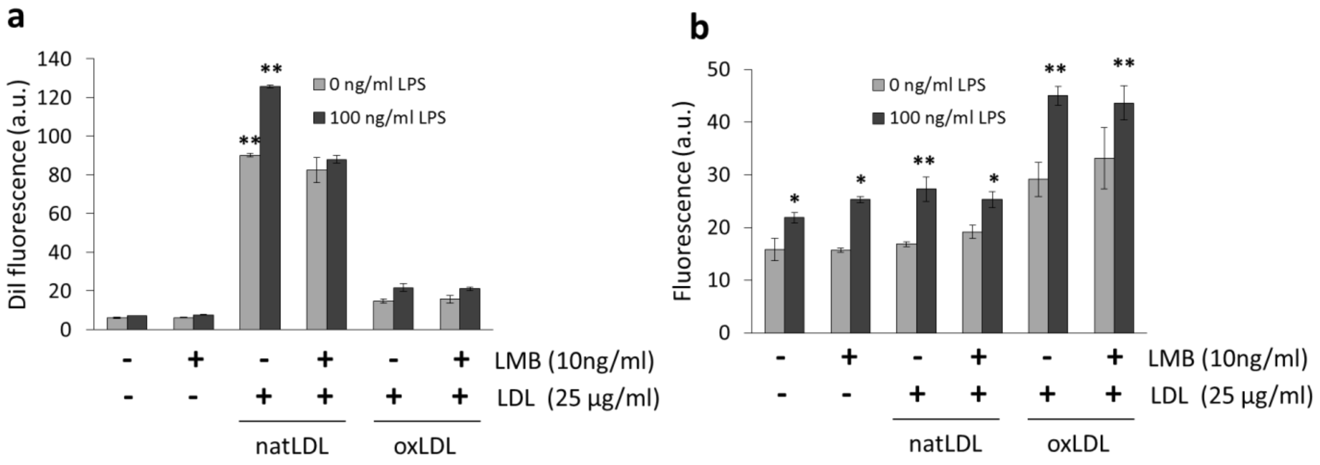

Supplementary fig. 4 | SR-B1 expression under treatment with oxLDL (10 µg/ml) in hepatocytes (**a**) and in macrophages (**b**).

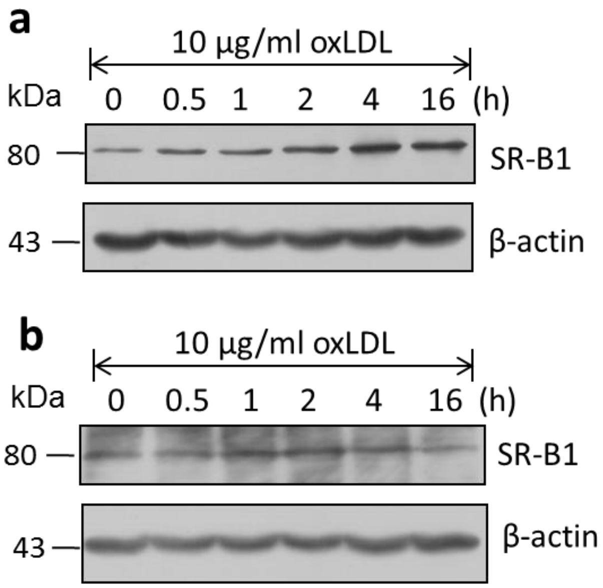

Supplement: Supplementary file 1 — Supplementary material [file 41598_2017_13880_MOESM1_ESM.pdf]
